# The Item versus the Object in Memory: On the Implausibility of Overwriting As a Mechanism for Forgetting in Short-Term Memory

**DOI:** 10.3389/fpsyg.2016.00341

**Published:** 2016-03-10

**Authors:** C. Philip Beaman, Dylan M. Jones

**Affiliations:** ^1^Centre for Cognition Research, School of Psychology and Clinical Language Sciences, University of ReadingReading, UK; ^2^School of Psychology, Cardiff UniversityCardiff, UK

**Keywords:** auditory cognition, short-term memory, memory, forgetting, auditory scene analysis

## Abstract

The nature of forgetting in short-term memory remains a disputed topic, with much debate focussed upon whether decay plays a fundamental role (Berman et al., 2009; Altmann and Schunn, 2012; Barrouillet et al., 2012; Neath and Brown, 2012; Oberauer and Lewandowsky, 2013; Ricker et al., 2014) but much less focus on other plausible mechanisms. One such mechanism of long-standing in auditory memory is overwriting (e.g., Crowder and Morton, 1969) in which some aspects of a representation are “overwritten” and rendered inaccessible by the subsequent presentation of a further item. Here, we review the evidence for different forms of overwriting (at the feature and item levels) and examine the plausibility of this mechanism both as a form of auditory memory and when viewed in the context of a larger hearing, speech and language understanding system.

A key feature of the classical short-term memory (STM) research program is the importance of serial order (Lashley, 1951; Conrad, 1960; Murdock, 1968, 1983; Lewandowsky and Murdock, 1989; Henson, 1998; Brown et al., 2000; Botvinick and Plaut, 2006; Burgess and Hitch, 2006). Words, letters, or digits are presented sequentially and participants required to recall the items in the order in which they were presented. Short-term memory tasks are usually deliberately designed so that the associations to be held across multiple items (words, digits, letters) are arbitrary. Performance in such tasks is framed in terms of its proximity to verbatim recall of all the items, namely the correct item in its position at presentation. Implicitly, short-term memory theorists make the assumption that the “item” (the word, letter, or digit of interest)—rather than the relationship between items—is the most meaningful unit of analysis. Moreover, identification of the item at recall is the basis for correct scoring in the memory test. Despite known problems with identifying the “item” in anything other than a logically circular way (Miller, 1956) such an approach is defensible in cases where the items are well-known and taken from a small, circumscribed set (e.g., digits) and where recollection of an item at a time collapses into a requirement to select the most likely candidate given the degraded or incomplete information available (Nairne, 1990). This contrasts starkly with the situation in most everyday language, in which structured, non-arbitrary relationships are available between individual elements represented at multiple levels (phonotactic, syntactic, semantic, pragmatic) and the identification of a single “item” is neither necessary nor sufficient to comprehend the meaning of the sequence.

By considering veridical recall of arbitrary items rather than the relationships between them, much of interest is lost with regards to later analyses. A key component of perception is in organizing as well as registering information and of interest is whether, in registering and organizing the stimuli prior to retrieval, the perceptual system represents them in a way that harmonizes with the retrieval requirements in standard short-term memory tasks. Given the emphasis on the iterative retrieval of items across the to-be-remembered sequence, does the perceptual system, for instance, cluster items at a supra-item level in such a way as to aid or to hamper efficient retrieval? In other words, does perception result in ‘items’ corresponding exactly to items specified in terms of the linguistic taxonomy (such as single syllables, words, or digits) on which the sequence is nominally based? In the event of supra-item organization, how are items grouped or transformed? Is there grouping of adjacent elements (as with chunking, classically) or are non-adjacent items organized into a greater whole? Within item-focussed approaches to short-term memory—ones that assume recall is a product of an aggregation of elemental actions—forgetting may be explained by the ‘overwriting’ of items by subsequent events. If supra-segmental organization occurs, is overwriting still a plausible mechanism?

Here, we explore how the registration of events in memory reflects auditory input and, in particular, the organizational processes that are at play. On the basis of key phenomena in auditory perception we consider potential implications for the structure of short-term memory and, in particular, the nature of forgetting.

## The “Standard” Model of Memory

The modal model of memory, informed by neuropsychological case data, has always assumed a functional and structural distinction between short-term and long-term memory, with the former fed by largely unspecified perceptual input processes, frequently depicted as a buffer storage system (Shallice and Cooper, 2010). In long-term memory, where the notion of memory as a reliable, veridical system has long since been dismissed and a reconstructive account of recall is generally accepted (Bartlett, 1932), suppression, inhibition and blocking of the memory trace have all been discussed as possible explanations of forgetting (for example in the context of the misinformation effect in eyewitness memory). In contrast, discussions of short-term and sensory memory have been less open to the idea of memory distortion as normal and recall as a reconstructive activity. In consequence, processes that highlight deterioration of the representation such as decay and overwriting (respectively) have predominated as mechanisms for forgetting and active supra-segmental organizational processes (such as grouping into objects), which may equally hamper recall when they are inconsistent with retrieval requirements, have been largely ignored.

Much has already been written both critiquing the evidence for decay (e.g., Neath and Nairne, 1995; Nairne, 2002; Lewandowsky and Oberauer, 2008, 2009, 2015; Lewandowsky et al., 2008; Oberauer and Lewandowsky, 2008, 2013; Brown and Lewandowsky, 2010; Neath and Brown, 2012) and defending the concept (Altmann and Gray, 2002; Portrat et al., 2008; Altmann, 2009; Barrouillet et al., 2011; Altmann and Schunn, 2012) so, rather than repeating now-familiar arguments about decay versus some other (often unspecified)^1^ form of interference as the source of forgetting (see Ricker et al., 2014, for a review), here we will specifically consider interference by overwriting as it appears from the perspective of auditory perception and the organization of the auditory environment.

The introduction of overwriting or displacement as a key determinant of forgetting over the short-term can be traced back to early studies of auditory sensory memory. Classically, a restricted-capacity acoustic sensory memory trace, overwritten by subsequent auditory events (Crowder and Morton, 1969), is available to supplement end-of-sequence recall otherwise only supported by “post-categorical” short-term memory systems dedicated to verbal memory but otherwise blind to sensory modality or the perceptual origins of the memoranda. This venerable account is nonetheless still extant and has been incorporated into more recent formulations of the contribution of sensory memory to immediate recall of auditory-verbal material (e.g., Page and Norris, 1998, and Burgess and Hitch, 1999, both make reference to an auditory input buffer overwritten by subsequent data). Latterly, other formulations of short-term memory have also utilized overwriting as a means of implementing interference and hence forgetting. For example, in providing a framework for short-term memory that eschews decay as a concept, Nairne (1990, 2002), Neath and Nairne (1995), Neath (2000), Oberauer and Kliegl (2006), Oberauer and Lange (2008), Lewandowsky et al. (2009), and Oberauer (2009) explicitly replace decay with overwriting as an explanatory concept. For memory of specifically auditory origin, therefore, three claims have been made:

(1) An auditory sensory store is overwritten, an item at a time, during encoding (e.g., Crowder and Morton, 1969; Page and Norris, 1998; Burgess and Hitch, 1999; Mercer and McKeown, 2010a).
(2) Both modality specific (sensory) and modality-independent (post-categorical, phonological) features are overwritten during encoding (e.g., Neath and Nairne, 1995; Neath, 2000).
(3) Features are overwritten via neural competition removing the availability of feature-units at a post-encoding phase (Oberauer and Kliegl, 2006; Oberauer and Lange, 2008; Oberauer, 2009).

It is interesting that the “precategorical” acoustic nature of the auditory sensory store (Crowder and Morton, 1969) arose because of prior theoretical commitments to a model of word recognition—the logogen model—which assumed a single system for recognizing both written and spoken words (Morton, 1964, 1969). Subsequent to this, changes to the logogen model (Morton, 1979) removed this theoretical constraint and introduced separate auditory and visual input logogens so that the idea that overwriting occurred at an early processing stage was retained even though the original *a priori* reasons for assuming that overwriting occurred prior to word-identification had vanished. The Crowder and Morton (1969) view is, despite its commitment to a pre-categorical (presumably continuous) representational format a classically item-based data buffer system of the first-in, first-out variety. Their approach can be contrasted with the forms of overwriting implemented in models by Nairne (1990) and Lange and Oberauer (2005).

In Nairne’s (1990) feature model of immediate memory, individual items are represented as vectors of features, which may represent modality-specific or modality-independent information. The eponymous features were speculatively identified with patterns of neural firing by Beaman et al. (2008) and, although their exact nature and status has never been formally defined, it is at this level that overwriting operates within the model. Feature overwriting works by an incoming item deleting identical features already held as part of the representation of immediately preceding item. For example, if the third feature of item *n*+*1* of a sequence takes the same value as item *n* of the same sequence then the item-level representation of *n* is denuded of this feature, the representation becomes degraded as a consequence and *n* is henceforth less likely to be correctly recalled when cued to do so at some point in the future.

In contrast, the version of overwriting put forward by Lange and Oberauer (2005) and Oberauer and Kliegl (2006) interference is not limited to the preceding item. Like the approach of Nairne (1990), the model is once again feature-based; in this instance, however, different items are represented as patterns of activation across a subset of the features (“feature units”) available system-wide and representations compete for access to their constituent feature units. Where a given representation loses this competition, the feature unit is captured by that competitor and is not available as part of the item representation of the “losing” representation. In this way a particular representation is degraded, thus impeding recall. The neural competition for features is framed in terms of synchronized firing of neurons as a mechanism of binding together the features that belong to the representation of an item (Raffone and Wolters, 2001). Feature units possessing features belonging to the same representation fire synchronously, whereas units belonging to different representations fire out of synchrony.

The principal difficulty with overwriting as the sole, or key, determinant of failure to recall in these or any other accounts is that while many studies have reported greater interference when irrelevant information (e.g., from a secondary task; Lange and Oberauer, 2005) is related to the memoranda, or when the list items are themselves similar along a specific dimension (e.g., the phonological similarity effect; Conrad, 1964; Conrad and Hull, 1964; Baddeley, 1966) other studies have shown the opposite. Overwriting in the three accounts given above assumes that interference occurs between similar items or items with similar features – acoustic items displace earlier acoustic items in a precategorical store (Crowder and Morton, 1969) or features are overwritten if they are shared between successive items (Nairne, 1990) or if they are supported by common feature units (Lange and Oberauer, 2005). These assumptions readily account for data in which interference is observed at recall between items that are similar along one or more crucial dimensions. However, Mercer and McKeown (2010a,b) found that complex tones were more accurately identified in a same-different task when followed by distractors containing *novel* frequencies – those frequencies not present in the target - when compared to a condition in which the distractors shared frequencies with the target. This pattern of results is directly contrary to that which would naturally occur if similarity-based overwriting was in operation.

Interestingly, Mercer and McKeown (2010b) also concluded in favor of an overwriting account – but in their model, directly contrary to assumptions made by other theorists about overwriting, “interference is principally caused by tones that include *novel* features since these will be most potent in “overwriting” the contents of the auditory spectral short-term memory buffer” (Mercer and McKeown, 2010b, p. 1258, emphasis added). In other words, this model assumes overwriting by items which are representationally distinct from the preceding input, rather than by items which share features with earlier items. Whether overwriting is assumed to occur amongst similar or dissimilar items/features is, of course, an *a priori* decision for any theorist attempting to construct a model (Lewandowsky and Farrell, 2011) but it is unlikely that similar items would be overwritten in some cognitive systems and dissimilar items overwritten elsewhere. To allow that closely related cognitive and perceptual subsystems work on diametrically opposed principles is, at best, un-parsimonious and contrary to Occam’s Razor. If overwriting is to be accepted then a consistent set of rules should apply (Surprenant and Neath, 2009). Nor is the study by Mercer and McKeown (2010b) (which involved fairly “low-level” and non-verbal acoustic stimuli) unique in its findings. An earlier study by Nairne and Kelley (1999) showed that the phonological similarity effect observed with verbal stimuli is reversed after relatively brief periods of distraction, resulting in better performance in a serial order reconstruction test for phonologically similar lists than for phonologically dissimilar lists. If overwriting is seen as necessary to account for forgetting caused by interference effects between similar items, then reversing these similarity effects casts doubt upon the need for overwriting.

Finally, task requirements—which are unlikely to directly influence low-level processes such as overwriting/displacement of patterns of neural firing or competition for neural feature units—also play a substantial role in similarity effects for which overwriting is offered as an explanatory mechanism. Despite numerous documented similarities between immediate free and serial recall (Beaman and Jones, 1998; Bhatarah et al., 2006, 2008; Ward et al., 2010; Grenfell-Essam and Ward, 2012; Grenfell-Essam et al., 2013; Spurgeon et al., 2014) similarity effects within the to-be-recalled list —supposedly reflecting the impact of item-representations degraded by direct over-writing (Nairne, 1990) or competition for specific feature units (Oberauer and Kliegl, 2006)—depress performance on immediate serial recall tasks but enhance performance in free recall (Fournet et al., 2003). Once again it is difficult to reconcile such findings with a low-level, item-based and automatic overwriting interference process without appealing to a higher-level activity that negates, and more than negates, the negative effect of similarity-based overwriting. To account for the reversal of the phonological similarity effect, Nairne and Kelley (1999) proposed that a period of distraction allows phonological similarity to be used as a cue to select candidate items for serial reconstruction of order (e.g., any correct item must share a rime with all other items) and similar suggestions are equally applicable to free, or item, recall situations (e.g., Watkins et al., 1974; Saint-Aubin and Poirier, 1999). However, such accounts are necessarily *post hoc* and—if overwriting occurs—strategies such as these must be sufficiently ubiquitous and powerful enough to not only negate but *reverse* the similarity effects otherwise observed. Exigencies of space mean that the interesting issue of retrieval mode and streaming cannot here be addressed fully but we note that free recall is in part controlled by strategic retrieval factors so that we may expect effects such as those of similarity to be different dependent upon the mode of recall and, critically, scoring technique employed. A stream of similar-sounding items will necessarily lose order cues relative to a dissimilar stream up until the point that items become so dissimilar that stream coherence is lost (Jones et al., 1999). There are no such necessary consequences for retrieval of individual items so scoring criteria at test are crucial in the appearance and form of similarity effects.

## Auditory Scene Analysis: Some Preliminaries

If a structural account of forgetting is set aside, what remains? Perceptual organization has profound consequences not just for the coherence of our experience of the world but also for the accessibility of information contained within it. Perception itself is directly linked to memory, as, for example, the perception of loudness is determined by a temporal integration of acoustic power; the perceived loudness of a burst of white noise depends upon its duration (Scharf, 1978) demonstrating that perception is reliant upon memory in a manner which renders the simple idea that incoming stimuli “automatically” overwrite pre-existing representations problematic. There is a mass of evidence showing powerful effects of perceptual organization and, as with vision, it is useful to think of auditory perception in terms of objects. So, despite being intrinsically evanescent in a way that the visual world is often not, successive events are assembled into temporally extended objects in a way that allows several “streams” of information to co-exist. Note that this is immediately different from the situation assumed within most models of verbal STM, which concentrate upon memory for a single list, and require further work to allow simultaneous representation of multiple lists or streams within the same representational space. Generally, the rules of organization follow Gestalt principles that are based on the physical attributes of the stimuli: proximity, similarity, closure, symmetry, common fate, continuity, among others. So, auditory perception is an active process that partitions the auditory world into auditory objects or streams, a process known generically as *auditory scene analysis* (Bregman, 1990). It is difficult to overstate the importance that these forces of organization have on what may be retrieved from an auditory scene, even when the scene comprises a few simple stimuli.

Necessarily, stream formation involves memory. A succession of individual stimuli achieves stream quality by a process that depend on not just a single but many preceding stimuli, a process that requires storage. Streams take time to form and less compelling streams can vacillate and break down. In everyday environments, scene analysis typically results in several simultaneous streams, such as the instruments of a rock bank or orchestra, or indeed a domestic scene of refrigerator noise, radio and conversation. Also, the principles by which this is achieved are embodied in musical polyphony: the rules of composition—though in a non-acoustic language—allow a composer to generate an intelligible and coherent rendition of harmonic and melodic intent.

So, the logic adopted here is that auditory memory is intimately connected to auditory perception and that in turn the study of auditory perception suggests ways in which auditory-verbal memory is organized. Furthermore, we know that this organization is not veridical, in as much as it does not faithfully represent an item-by-item sequence, free of item clusters. As we will see later, the item-clusters produced by auditory perception are very much richer and more diverse than those considered by current models of verbal STM.

**FIGURE 1:**
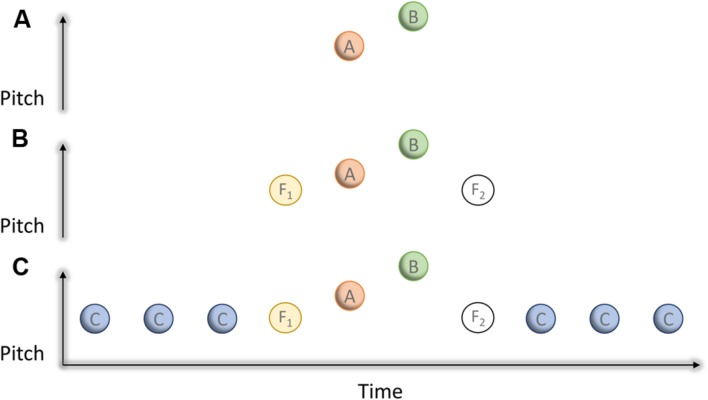
**Arrangement of stimuli in the experiment of Bregman and Rudnicky (1975).** **(A)** Shows two stimuli—A and B—differing slightly in pitch. **(B)** Shows the case where two stimuli (flankers) of identical pitch—F1 and F2—flank the AB pair. **(C)** Shows the case in which a sequence of stimuli—the captor C stimuli—precede and follow the flanker stimuli. The flankers and captors share pitch and timing.

It is useful to consider specific instances, using some very simple non-verbal stimuli, of how perceptual organization of sound brings about changes to perception before returning to the case of verbal memory. The first example shows how the context in which stimuli appear works to shape what we may know of them. Take the very simple case of two short tones, A and B, the same in every respect except that they are a semi-tone apart, presented in quick succession (see **Figure 1**). When faced with the task of reporting the order as being high-low or low-high, most listeners find they can make the discrimination quite easily. However, if flankers (F_1_ and F_2_)—sharing almost the same pitch and tempo as A and B (see again **Figure 1**)— are inserted either side of them then we observe a dramatic reduction in the capacity to report the order of A and B. How might this come about? One way to think in terms of overwriting and to suppose that the second flanker (or indeed both flankers) somehow interfere with the representation of A and B, making their comparison less easy. Another way is frame the change in context in terms of object formation. Whilst presented as a pair, A and B formed a single object and at the same time constituted its boundaries. Adding the flankers created a new object and new boundaries, with A and B now constituting its innards, so that now the order information contained in A and B becomes more difficult to address. This is a familiar situation in STM where current recall of the items and order of the first and last few items gives rise to primacy and recency effects, with recall of items in the correct order very much worse toward the center of the list.

A simple further addition to this auditory scene shows how implausible the overwriting explanation turns out to be in the case of simple tones. If we add a further two stimuli (C_1_ and C_2_ in **Figure 1**) either side and sharing both pitch and tempo with the flankers then we witness a remarkable transformation: if we now ask a listener to judge the pitch order of A and B, close to full efficiency (that is, the level of performance when A and B are presented in isolation) is restored. Clearly, according to the overwriting view (and indeed, most interference theories of forgetting) adding more stimuli should – if anything – produce more overwriting, not less. However, the outcome of adding the C stimuli is readily understood in terms of auditory scene analysis. The C stimuli act as ‘captors,’ that is, by virtue of their greater similarity to the F stimuli than to the AB pair, two objects are formed; the one: CCCF_1_F_2_CCC, the other: AB. The flankers are captured, releasing AB to become a separate, and therefore an independently addressable, entity thereby restoring memory for the order of A and B.

This setting shows several remarkable qualities of auditory scene analysis with a number of important implications for the way we understand memory. The first and most profound is that the future shapes the past: perception is retroactive. What follows from this has great relevance to our current discussion about the plausibility of overwriting as an explanatory construct. Critically, the perceptibility of AB is only decided when both F_2_ and CCC are presented, but even then both F_2_CCC and AB are distinguishable only with reference to F_1_ and CCC. The first point that follows from this is that it is important therefore to think in terms of the emergent properties of the stimulus ensemble (the object), not merely as an aggregation of the properties of individual stimuli. The second point is that items need not be temporally adjacent in order to form into objects.

A second illustration lends weight to the first while at the same time addressing the natural skepticism that such a simple setting involving the mere ‘perception’ of tones A and B could have more general repercussions for more complex settings that we think as being characteristic of the study of ‘memory.’ Here again, the listener is asked to compare two tones but this time asked to make the judgment about whether they are the same or different in pitch (Jones et al., 1997).

**FIGURE 2:**
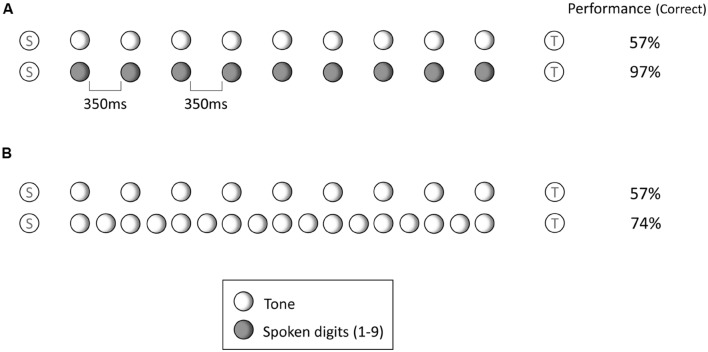
**The stimuli used by Jones et al. (1997).** Both parts of the figure show an arrangement of tones both each with an initial standard tone (S) and final test tone (T), but with different interpolated sequences. Participants are asked to make a ‘same’ or ‘different’ judgment in terms of the pitch of the tones. Performance in terms of percent correct responses is shown on the right. **(A)** Shows two cases: one with interpolated tones and the other with interpolated spoken digits. **(B)** Shows the case of the standard rate of presentation and below the case where the number of stimuli is doubled.

**Figure 2** shows the arrangement of stimuli used by Jones, Macken, and Harries (following, for example, Deutsch, 1972, 1978a,b; Semal and Demany, 1991, 1993; Starr and Pitt, 1997; Mathias and von Kriegstein, 2014). First, a standard stimulus—a tone—is followed either by a blank interval or a filled interval and then, some seconds later, by a comparison stimulus: another tone. The listener is asked to ignore stimuli that come between the standard and comparison tones in making their judgment.

The key variable of interest is the content of the interval and its effects on the accuracy of the comparison judgment. Having a sequence of tones in the interval similar in pitch and timbre to the standard and comparison (see **Figure 2**) has a dramatic effect of reducing the accuracy of the same-different judgment. If, instead of having tones, we have speech stimuli (say a sequence of words), comparison judgment improves considerably, to a level that is close to when there are no interpolated stimuli. This result is conventionally interpreted in an overwriting framework: memory for the standard is compromised by similar stimuli interpolated between it and the comparison (e.g., Semal and Demany, 1991, 1993; Mathias and von Kriegstein, 2014). However, another of the conditions in the study of Jones et al. (1997) makes this interpretation implausible. If the number of interpolated tones is doubled then any reasonable interpretation the overwriting account suggests that performance cannot improve and should, in fact, deteriorate. If overwriting interferes only with the immediately preceding item (as with Nairne, 1990) then the level of interference remains the same, although the increase in the number of sources competing for consideration at recall could still negatively affect overall performance. If overwriting is not restricted to immediately preceding items (as with Lange and Oberauer, 2005) then performance should deteriorate, and appreciably so given the rise in number of interfering sources. In the event, the opposite turns out to be true; performance improves significantly.

If we construe the setting in terms of auditory scene analysis, this last result is entirely intelligible. In object terms, the proximity of the standard to the interpolated tones and the similarity of their physical character (sharing tone-like qualities), along with its shared timing, increases the likelihood that it will be incorporated with them into an object, thereby reducing its identity as a separate entity. When the interpolated material is speech, of course this tendency will be much less likely. Doubling the number of interpolated tone stimuli is likely to produce an outcome similar to that seen with interpolated speech stimuli: by virtue of shared timing (in addition to shared pitch and timbre) the interpolated stimuli will in this case form an object separate from the standard. The judgment of similarity is once again based on two stimuli distinct from the interpolated stimuli: the scene comprises three objects, a standard, a distinct interpolated stream, and the comparison.

Streaming thus produces important consequences for our judgment of the plausibility of overwriting as an explanatory mechanism and for hearing and memory. The context in which stimuli appear has powerful repercussions for what we can retrieve of stimuli. As we shall go on to consider, the fact that auditory stimuli appear in chronological order does not mean that that access to temporally adjacent stimuli is guaranteed. So, for instance, if we present a sequence in alternating male-female voices (M_1_F_2_M_3_F_4_M_5_F_6_), two streams are formed (M_1_M_3_M_5_ and F_2_F_4_F_6_) a situation that contrasts with a single (e.g., male) stream: M_1_M_2_M_3_M_4_M_5_M_6_. By forming two distinct streams it will become more difficult to retrieve chronologically adjacent stimuli (e.g., M_1_F_2_M_3_F_4_ will be harder to retrieve than M_1_M_2_M_3_M_4_), but easier to retrieve stream-adjacent (and chronologically non-adjacent) stimuli (e.g., M_1_M_3_M_5_ will be easier to retrieve in the alternating voices case) if cued to retrieve the utterances in strict temporal order of their occurrence. Notice that—as suggested earlier—the stream can contain non-adjacent elements. This contrasts with the typical interpretations of ‘chunking’ (and also “grouping”) that invariably refer to an aggregation of temporally adjacent elements. Auditory scene analysis shows that even quite remote elements may be assembled into an organized whole. This is why scene analysis and chunking are slightly different mechanisms and why it is important to consider remote elements in any scene analysis (see Jones, 1993, 1999; Jones et al., 1996; for extended discussions). This relates to the question of overwriting because temporally remote and non-adjacent items can have a greater effect upon memory for any given target item than does the immediately subsequent item, a result which is inconsistent with at least two forms of overwriting (Crowder and Morton, 1969; Nairne, 1990)

Perhaps the simplest and most telling prediction from the overwriting hypothesis is that sequences with fewer shared features should be easier to retrieve than those with many shared features. This follows from such ideas as the relative distinctiveness principle, the suggestion that an item (or series of items) perceived to be discriminable on some dimension(s) from its fellows is easier to recall by virtue of psychological distinctiveness (a principle which is consistent with overwriting as an underlying mechanism, although other mechanisms may produce such an outcome; Surprenant and Neath, 2009; Neath, 2010). Evidence already reviewed indicates that this is not always the case, and further data indicate that streaming may be a useful concept in explaining outcomes that run contrary to this principle.

Very distinct non-speech sounds, when presented quickly in a sequence are easy to recognize, so that listeners can judge they are present but are less able to indicate the order in which they appeared. So, if a sequence of very different sounds—a high-pitched tone, a hiss, a low-pitched tone and a buzz—are heard in a repeating cycle, listeners are able to name each of the sounds. However, they cannot report their order correctly, even if the period of listening is extended indefinitely (Warren et al., 1969; see also Jones et al., 1999). However, if a sequence of four spoken digits—spoken in the same voice—is presented under the same conditions, the order can be readily reported. The key difference between these two settings is in the level of commonality in acoustic content: acoustically the digits form a variation on a common ground and so quickly form a stream, but for the non-voice sounds each element constitutes a separate entity and streaming is less easy to achieve.^2^ Consider how such a situation would be addressed by Nairne’s (1990) feature model, in which automatic overwriting forms a large part. The identity of the stimuli themselves would be represented in secondary memory, so the task would simply be to match the correct primary memory representation to the correct secondary memory identity in the correct order. The task would be made difficult by the fact that overwriting would degrade the primary memory representations such that the primary-secondary memory match would become more problematic and, potentially, confused. This confusion would clearly be more prevalent in the situation under which the most overwriting occurred – when the stimuli come from a common source (spoken digits) and share common acoustic and lexical features. These results pose grave difficulties for an overwriting account; distinct sequences should be subject to less overwriting, but the results are diametrically opposite. The explanation comes from stream formation: when the stimuli are perceived as originating from a common source they form a single stream within which order is preserved.

## Auditory Scene Analysis: The ‘Suprasegmental’ Approach Applied to Verbal Memory

In view of the problems outlined earlier, we wish to outline an alternative framework in which retrieval (in both sensory and short-term memory) is constrained by perceptual principles. The primary line of argument we wish to pursue is that the need to maintain a coherent stream of information over time constrains the processes operating within memory and hence automatic and immediate overwriting of an item representation or the features representing an item is not a tenable explanation for forgetting. The auditory scene analysis principles outlined above, however, were introduced with reference to simple auditory stimuli (e.g., tones) and in what follows these are expanded to encompass more traditional verbal memory phenomena.

Within auditory memory, overwriting was originally proposed as an explanation for the interference associated with a post-stimulus suffix (Crowder and Morton, 1969) so we turn first to this phenomenon and possible alternative accounts.

### The Failure of Overwriting: Capturing the Suffix

Classically, the existence of acoustic storage termed the “precategorical acoustic store” (PAS; Crowder and Morton, 1969) was assumed to precede “post-categorical” verbal storage (where modality of origin – spoken or written – is irrelevant, a common abstract representation is shared by all stimuli, regardless of input modality). Its existence was inferred from the auditory recency effect, in which the final item of an auditorily presented list for serial recall is recalled at near-ceiling levels compared to the much smaller recency effect obtained with visually presented lists of the same verbal items. The reason that this has been attributed to a restricted capacity acoustic store is that elsewhere along the list performance on visually and auditorily presented lists is broadly equivalent (but see Beaman, 2002; Macken et al., 2015) and is affected in a similar manner by standard verbal manipulations such as phonological similarity, word-length, and concurrent articulation. The final piece of evidence provided in support of PAS was that the presence of a post-stimulus suffix effectively eliminates this final-item advantage, leading Crowder and Morton (1969) to conclude that the stimulus suffix effect “depends upon selective displacement of information from PAS” (Crowder and Morton, 1969, p. 369).

Crowder and Morton (1969) assumed an item-by-item displacement system rather than feature-based overwriting and one reason for disputing the feature-based interference account of the stimulus suffix effect comes from data showing that stimulus suffixes which are phonemically similar to the memoranda do not necessarily show larger suffix effects (Crowder and Cheng, 1973) and—like the other similarity-based interference effects already reviewed—may also show smaller effects (Carr and Miles, 1997). Another reason for questioning feature-based overwriting comes from studies of *streaming* the suffix. It is well established that the stimulus suffix effect depends at least in part upon the suffix being perceived as originating from the same sources, or stream, as the to-be-recalled list. So, for example, variations in the spatial location, timbre and pitch of the suffix relative to the list reduces the size of the suffix effect whereas similar manipulations varying suffix frequency, emotionality and meaning have no such effect (Morton et al., 1971). Other manipulations varying the “speech-like” qualities of the suffix similarly moderate the size of the suffix effect (Morton et al., 1981). Manipulations of the top-down interpretation of the suffix likewise show that forcing the suffix to be grouped with, or apart from, the list affects the auditory memory interference effect (Crowder, 1971; Frankish and Turner, 1984; Neath et al., 1993). So, for example, ambiguous stimuli, which can be perceived as either speech or non-speech, can be treated as a speech suffix on the basis of labeling them as such (Ayres et al., 1979; Neath et al., 1993). However, other non-speech stimuli do not show a suffix effect unless contextual effects also force them to be perceived as speech (Morton and Chambers, 1976; Ottley et al., 1982). These results show that physically identical stimuli, which bear physically identical relationships to the memoranda, can produce different memory effects depending upon context and expectation. At best, therefore, any interference effect obtained under such circumstances can only be ascribed only in part to the physical overwriting of the memory trace.

Perhaps most intriguingly, the effects of a repeated suffix have also been shown to reduce the disruption observed (Crowder, 1971, 1978; Morton, 1976). With a repeated suffix, the same suffix item is presented multiple times in quick succession and in tempo with the list sequence (as usually also happens with a single suffix). The reduced effect of the suffix when repeated in this way, even though the first presentation of the repeated suffix is physically identical to the presentation of a single suffix, is difficult to reconcile with an overwriting account based upon physical or feature similarity between successive items since the relationship between the suffix and the list items is equivalent in the two conditions. Critically, a repeated suffix only becomes a repeated suffix at the point of its re-presentation; logically, therefore, automatic overwriting occasioned by the first presentation of the suffix must already have occurred at this point. Data such as these have led to suggestions that the suffix effect might reflect the simultaneous action of overwriting, accounting for the reduced suffix effect still observed, and perceptual grouping, accounting for the difference between single and repeated suffix effects (e.g., Morton, 1976). According to these accounts, the repeated suffix forms a perceptual group apart from the to-be-remembered list whereas the single suffix is perceived as part of this list. It follows from this that the sole cause of the disruption observed in the repeated suffix condition is from overwriting. A single item suffix likewise overwrites the final item but also further depresses memory performance by increasing the functional size of the memory set (the list length) by an extra item (e.g., Nairne, 1990).

**FIGURE 3:**
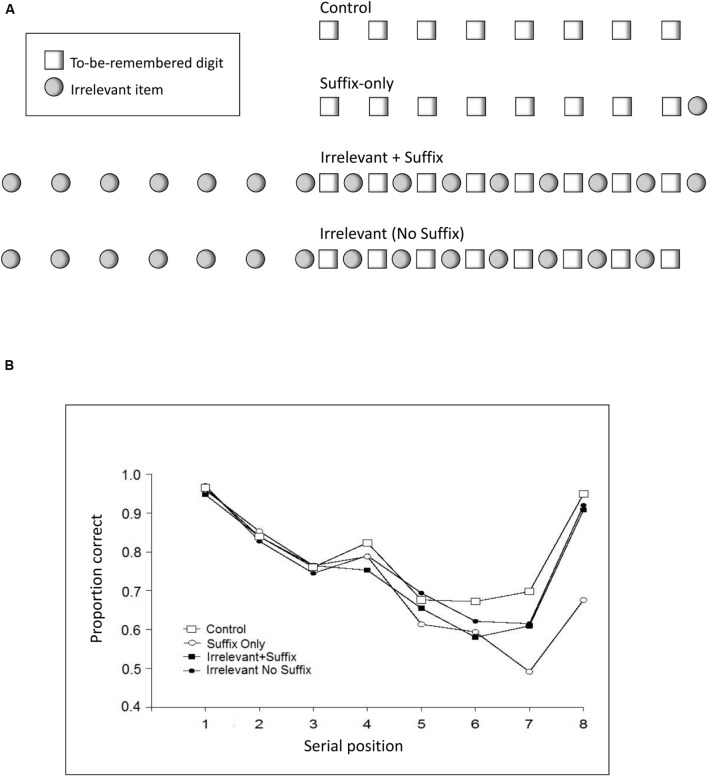
**(A)** Shows schematically the arrangement of stimuli used by Nicholls and Jones (2002). The control condition comprises a sequence of eight to-be-remembered digits. The suffix-only condition has a spoken irrelevant item ‘zero’ at the end of the to-be-remembered sequence. The Irrelevant + Suffix condition shows a sequence of irrelevant stimuli (‘zero’) beginning well before the irrelevant sequence and culminating with an item after the last digit in the to-be-remembered sequence. The ‘Irrelevant (No Suffix)’ condition is the same as the Irrelevant + Suffix condition without a terminal suffix. **(B)** Shows the performance associated with each of those conditions as a function of the presentation position of the stimuli within the to-be-remembered sequence.

These data undermine the importance of overwriting as the source of the suffix memory disruption effect but do not rule out the possibility that overwriting occurs; perhaps it is merely contributing only part of the observed disruption. Later data reported by Nicholls and Jones (2002) are, however, less equivocal. In their experiments, Nicholls and Jones (2002) interleaved a sequence of irrelevant items between the to-be-remembered list items such that the suffix, when presented, was perceptually grouped with, or “captured” by, these irrelevant items. The sequence comprised the item ‘ah,’ which was also used as the suffix in a traditional suffix effect condition (see **Figure 3**). When no-suffix, suffix and captured suffix conditions are compared, it is clear that in the captured suffix condition performance approximates that to the no-suffix condition^3^. In the captured suffix condition the recency effect was fully restored and there was no suffix effect on the final list-item when the suffix was grouped, or streamed, with the sequence of irrelevant items. In contrast, the suffix presented alone continued to produce a suffix effect. Unlike the repeated suffix manipulation which reduced but did not eliminate the suffix effect, these data cannot easily be explained by the joint operation of overwriting and grouping since—in this case—the grouping (or streaming) manipulation removed the suffix effect entirely and hence the need to assume overwriting as the basis of the suffix effect.

**FIGURE 4:**
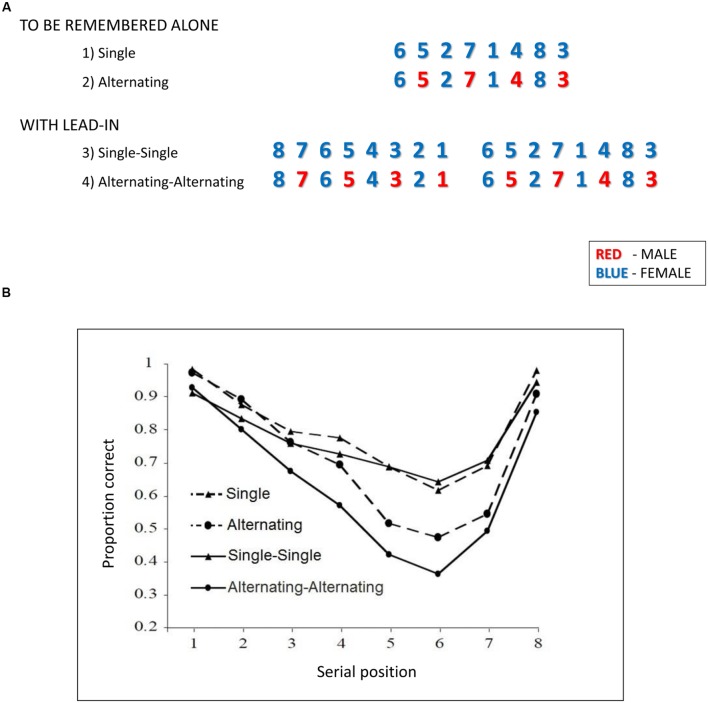
**(A)** Shows a sub-set of stimuli used by Hughes et al. (2009). To-be-remembered stimuli are first shown in isolation with lists either all from the same voice (Single) and then shown with alternating male and female voices (Alternating). Participants are required to report all the list in the sequence in which it was presented. Then lists with lead-in are shown. In the first case both the lead-in and the to-be-remembered list are in the same voice (Single–Single) and then in alternating voices (Alternating-Alternating). **(B)** Shows the performance associated with each of those conditions as a function of the presentation position of the stimuli within the to-be-remembered sequence.

Thus, the proposition that auditory-sensory memory is necessarily automatically overwritten is untenable. However, the suffix effect is only a single line of evidence. Recently, doubts about overwriting have been reinforced by findings from a paradigm using alternating voices for each list item and observing the consequences for memory of streams created in this way (Hughes et al., 2009). A suffix presented in a different voice reduces the suffix effect (Morton et al., 1971), consistent with the idea that overwriting depends on similarity between the suffix and the final list item but also consistent with the idea that a different voice suffix is grouped apart from the list items. If overwriting is automatic and based solely upon such physical properties and relationships between successive items, then presenting the to-be-recalled list in alternating voices (e.g., male-female-male and so on), should limit the overwriting observed between successive items compared to the same items presented in a single voice because the feature similarity is reduced by the voice change. Hence, overall recall should be enhanced relative to single-voice presentation. Alternatively, if perceptual organization is important so that items presented in different voices are streamed as coming from distinct sources, then recalling the items in the correct serial order should be harder. As noted in early research on auditory attention, items are preferentially recalled according to the stream or channel from which they are perceived to originate (e.g., Broadbent, 1958; for an extensive discussion see Hughes et al., in press) such that if the two voices are perceived as two separate streams then to recall the items in correct serial order requires participants to shift alternately between streams in order to reconstruct the serial order of the list. This extra cognitive requirement imposes a behavioral cost such that a list of alternating voices is not recalled as well as the same items presented in a single voice (Hughes et al., 2009; see **Figure 4**). Again this talker-variability effect calls into question the predominance of overwriting, which would predict the opposite pattern of results.

### Time, Space and Voice-Based Grouping Effects

The talker-variability effect, together with the different-voice suffix effect, supports the assumption that lists presented in different voices are perceptually grouped apart and that this influences the appearance of memory phenomena. Such assumptions find further support from early work on auditory attention (Broadbent, 1958) together with current theories of low-level auditory perception, within which auditory stream segregation (Bregman, 1990) plays a central role. One further line of evidence, however, serves to emphasize the relationship between perceptual organization and what seem superficially to be wholly mnemonic processes (suffix and talker-variability effects).

Work on grouping within auditory memory by Frankish and Turner (1984), Frankish (1985, 1989, 1995) directly examines the effect of perceptual grouping on subsequent recall. In a series of experiments, Frankish (1985, 1989, 1995) demonstrated that coherent groups can be formed within lists presented for immediate serial recall. These groups are defined by boundaries that exhibit the same, or similar, primacy and recency effects at recall as the longer lists of which they form a part. For example in a control (ungrouped) list, recency occurs only at the end of the list. However, in a 9-item list which is organized into three groups of three items each—for example by a delay in presentation between items 3 and 4 and between items 6 and 7—recency is seen for the final item of group 1 (at serial position 3, which must therefore be relatively immune to the suffix effects of item 4). Grouping is effective when it employs exactly those principles of perceptual organization important for reducing the suffix effect. These principles include change of voice, delay in presentation, and change of spatial location, all of which have been confirmed as producing within-list recency effects associated with groups (Frankish, 1989). The principles of grouping in auditory-verbal memory, it appears, are readily inferred from the data showing a reduction of the suffix effect. Additionally, Frankish (1985) showed that, with *visual* presentation, there is little extra grouping advantage by inserting extra pauses after the third and sixth items in the nine-item list. Frankish (1985) found no obvious difference between the serial position curves produced when participants are asked to subjectively group visual lists and those produced when the presentation of the lists was grouped by half second pauses (Experiment 1).

In a further study, Frankish (1989) showed that an extra pause of only 80 ms following the third and sixth items had as much effect as an extra half-second pause. Likewise, when the middle three digits were differentiated from the others by either voice (male vs. female) or spatial channel (left vs. right ear), the effects of these manipulations were equivalent to those of the temporal change. In addition, the study demonstrated that the voice distinction alone is as effective as voice plus pause. That is, if the middle three digits are in a different voice from the first and last three, then inserting a pause of half a second after the third and sixth digits, thereby, in addition, temporarily isolating the middle three digits, has no further effect.

These effects appear to reflect the automatic segmentation of auditory lists in a manner that is more powerful than the strategic grouping that operates on visually presented lists which produces less of an effect and is more readily disrupted (Hitch et al., 1996). Although a number of researchers (e.g., Hitch et al., 1996; Farrell, 2012) have concentrated on the role of timing—and of extended pauses—in creating groups, Frankish’s results clearly show that perceptual groups can be created using cues other than elongated pauses between list items. This observation is important because it shows that factors other than consolidation and rehearsal of a recently completed group (in the pause before the next group arrives) are responsible for creating these group boundaries. It also shows that the group boundaries can be established very quickly – parsing the list into subgroups almost instantaneously as the stimuli are encountered. Thus, although providing temporal cues to grouping and allowing (or encouraging; Taylor et al., 2015) prosodic, group-based rehearsal to emerge is one means of parsing the input, it is not the only way in which within-list organization can emerge. Crucially for current purposes, the perceptual segmentation of auditory lists is one that requires the constant comparison of the current and preceding auditory input. Automatic overwriting of previous stimuli by incoming information would interfere with the allocation of the current (incoming) stimulus to the appropriate perceptual stream, which may have been established over several preceding items.

### Principles of Organization: Similarity-Based Streaming

Generally, theories of short-term memory memory fail to acknowledge (or at most, pay lip-service to) the idea that events might be organized—and re-organized—according to perceptual streams. Rather, current theories view short-term memory as post-categorical, item-based encoding within a single, to-be-recalled list. The item here is defined by the experimenter *a priori* rather than inferred from the behavior of the participant. Those characteristics of the stimuli that denote common origin, that connote streams—among them similarity of pitch, timbre, location, and proximity in time—are ignored by such accounts, which also overlook the fluidity and flexibility of systems within which items are organized—and re-organized—according to their perceived belonging to one or more sources of origin. We argue that this is a profound mistake.

In the first instance, it is logical to assume that whatever form representations take in memory is constrained by the way in which information is available perceptually. The existence of natural organizational principles, known since the advent of Gestalt psychology, implies that multiple streams of information co-exist within memory in a way that is inconsistent with strict overwriting as the mechanism for forgetting. In the second instance, treating memoranda as discrete and independent items within the experimental participants’ cognitive systems because they were conceived and presented as such by the experimenter is an unwarranted assumption. The assumption arises directly from the idea that representations are, almost by definition, abstract and “post-categorical,” whereas in fact very few studies have examined the extent to which memory results can be accounted for by categorical vs. *continuous* storage systems (Frankish, 2008; Joseph et al., 2015). Taken to the extreme, it is clear that the recall of individual items is not independent, and whilst few models make this mistake, the amount and type of information relating the experimenter-defined items to one another and to a perceived locus of origin is impoverished in current theories. The relationship between items is formally often one merely of time or position (e.g., Page and Norris, 1998; Brown et al., 2007). Commonality of perceptual characteristics rarely plays a role because all of the elements within the memoranda are automatically assigned to a single list-structure, something that presumably occurs at a pre-mnemonic processing stage. Where between-item similarity is considered (as for example, to model the phonological similarity effect) this may often be at a distinct stage from positional similarity. For example, the primacy model of Page and Norris (1998) in which positional errors between localist representations occur naturally along the “primacy gradient” then forward items onto an explicitly phonological distributed representation stage prior to output in order to implement item confusion errors (Beaman, 2000)^4^.

Missing from all of these accounts is any measure of *stream-based similarity* such that elements within the memoranda are allocated to one stream or another based upon a common theme or thread running through the sequence and which serves to distinguish this stream from another. Stream-based similarity, according to this analysis, is necessary to account for the effects reviewed above – the reduction or elimination of suffix effects, the talker variability effect, the perceptual grouping effect and so on. The thread of similarity that acts to hold elements together is, however, precisely the source of interference that would consistently and continually degrade individual item representations under an overwriting account.

The availability of information about the stream to which the stimuli belong is precisely what is needed to account for moderation and abolition of suffix effects, between-talker variability effects, and within-list grouping effects as reviewed here. Discontinuities in time (i.e., elongated breaks between groups) have been used to account for within-list temporal grouping effects (Nairne, 1990; Hitch et al., 1996; Farrell, 2012). This mechanism follows naturally from the idea of overwriting, since a break is naturally interpreted as a pause in which information can be consolidated and/or within which retroactive interference (such as overwriting) will not occur. Such accounts do not properly address the effects of very short pauses between groups which are more parsimoniously conceived of as groupings caused by discontinuities in rhythm rather than time *per se*, nor are they able to account for grouping effects caused by intonation, timbre or spatial location. For the same reasons, speaker-variability effects and reduced suffix effects are not predicted by such accounts because the models do not maintain the correct types of information to give rise to such effects. To do so, not only must information about physical characteristics be maintained in addition to whatever post-categorical or more abstract labels that may be assumed, but also information must be held about the stream as a whole rather than individual items in isolation, and incoming information (e.g., a post-list suffix) interpreted in terms of the information held and prior expectations it elicits, as shown both by contextual suffix effects and by experiments repeating and streaming the suffix. The main conclusions point to the intimacy of perception and memory, or perhaps even to their wholesale integration. Certainly, no attribution to the action of auditory memory should be entertained until a thoroughgoin analysis of how auditory streaming could explain the same phenomena has been dismissed. Only after streaming processes have yielded the super-ordinate structure of the material being remembered can other approaches – such as overwriting – be entertained as explanatory constructs.

## Author Contributions

This manuscript was co-written by CPB and DJ. Figures were provided by DJ.

## Conflict of Interest Statement

The authors declare that the research was conducted in the absence of any commercial or financial relationships that could be construed as a potential conflict of interest.

## Footnotes

1 In fact, Lewandowsky et al. (2009, box 4) postulate at least four possible alternatives to trace decay and Ricker et al. (2014, Table 1) suggest five possibilities, all of which – as with decay itself – may be implemented in different ways (e.g., Lewandowsky and Farrell, 2011).

2 The rate of presentation in these studies is fast and this prevents verbal labeling; when the speed of presentation is slowed performance improves but only to a relatively small degree.

3 Notably, the mere presence of an irrelevant sequence of repeated items has no appreciable effect on serial recall.

4 This is a mirror image of how the feature model addresses the same situation: in the feature model, item-based confusions arise naturally from the distributed representation of items as vectors of feature values but positional errors only occur when an item independently “drifts” along the position dimension (Neath, 1999, 2000).
